# Short term outcomes of three dimensional versus two-dimensional laparoscopic cholecystectomy

**DOI:** 10.12669/pjms.37.1.3721

**Published:** 2021

**Authors:** Abdul Razaque Shaikh, Aijaz Ahmed Shaikh, Mujib Abbasi

**Affiliations:** 1 Dr. Abdul Razaque Shaikh, FCPS. Department of Surgery, Liaquat University of Medical Health and Sciences, Jamshoro, Pakistan; 2 Dr. Aijaz Ahmed Shaikh, MS. Department of Surgery, Liaquat University of Medical Health and Sciences, Jamshoro, Pakistan; 3 Dr. Mujib Abbasi, FRCS. Department of Surgery, Liaquat University of Medical Health and Sciences, Jamshoro, Pakistan

**Keywords:** Three Dimensional, Two Dimensional, Laparoscopic Cholecystectomy

## Abstract

**Objective:**

To compare the short-term outcomes of three dimensional (3D) versus two dimensional (2D) laparoscopic procedures used for cholecystectomy.

**Methods:**

This study was conducted at minimally invasive surgery center of Liaquat University of Medical Health and Sciences (LUMHS) Jamshoro Pakistan, between 15^th^ May 2017 to 16^th^ December 2017 after taking informed consent. All patients were diagnosed cases of cholelithiasis without any complications. Patients having risk factors for inability to get access to gall bladder via laparoscope and in whom the chances of conversion to open cholecystectomy were greater were not included as part of study. One group of patients underwent cholecystectomy under 3D laparoscopy while other group underwent 2D laparoscopy. Surgeons included in the study were all well-trained. The short-term outcome noted were intraoperative and postoperative complications, conversion to open, operative time, mortality and hospital stay. Visual strain and headache for the surgeon in three D laparoscopic cholecystectomy.

**Results:**

A total of one hundred forty patients were included in the study. Group-A consists of sixty two females and eleven males whereas Group-B comprised of fifty eight females and fifteen males. Eight percent of patients in Group-A whereas in Group-B two percent had gallbladder rupture. Fifteen percent of patients in Group-A whereas 5.4% from Group-B had bleeding from liver bed. One patient from Group-A had CBD (Common Bile Duct) injury. Post-operatively two (2.73%) patients from Group-A had port site bleeding. Six (8.21%) patients had port site infection in Group-A.

**Conclusion:**

Three dimensional was found to have low incidence of intra-operative and post-operative complications compared to 2D laparoscopic cholecystectomy.

## INTRODUCTION

Laparoscopic cholecystectomy is considered the gold standard operation for cholelithiasis due to shorter hospital stay, early recovery, decreased pain and decreased risk of infections. The advantages offered by laparoscopic surgery over conventional surgery include less damage to healthy tissue, reduced pain and risk of infections, as well as shorter recovery time and hospital stay. There are some haptic (touch) and visual problems (depth perception) in conventional 2 D laparoscopic cholecystectomy.^1^

It is often seen that in the natural environment, depth perception allows us to judge objects and their positions relative to our own bodies and from each other. In laparoscopic surgery (LS), the surgeon has no binocular imbalance when viewing the laparoscope with only one lens, and has limited vision distances, such as glossing, brightness and size. This poses major mental rotation and transformation challenges to the surgeon and should contribute to response delay, misjudgment, increased cognitive workload, and fatigue. Due to the greater cognitive processing demands, these consequences may cause intraoperative complications and unintentionally jeopardize patient safety. The need for better visualization was stressed further after the evaluation of 252 cases of cholecystectomy, where it was reported that 97% of surgical accidents during this procedure occurred as a results of visual misperceptions. Available technologies for presenting binocular disparity of MIS images include the 3D video monitor, the head mounted display, and the auto-stereoscopic display.^2^,^3^

The main disadvantages of two-dimensional (2D) laparoscopy are steep learning curves, lack of deep understanding and position. To overcome this deficiency, advanced three-dimensional (3D) laparoscopic systems was introduced with a stereoscopic view, in which deep vision is obtained by combining different images obtained by each eye. In 1990s, the first 3D laparoscope was invented on the phenomenon of shutter glass technique became available for use in clinical practice. Initially, surgeons were uncomfortable with the 3D system because of the heavy glass with active shutters, poor image quality and offered a visual burden on surgeons. Later on with the advancement of science, film type patterned retarder (FPR) was invented. This offers better images with minimal strain of surgeons’ eyes.^4^ In 2012, the introduction of FPR glasses by Buchs et al provided smooth images with better analysis if depth of structures.^5^ El Boghdady M et al reported Visual symptoms were present in both 2D and 3D imaging laparoscopy. Eye strain was prominent in 2D imaging, while difficulty in refocusing from one distance to another was prominent in 3D.^6^

There is no local study published in Pakistan but a regional study has been published from India.^3^ Moreover, the novices who are trained using the 3D laparoscopic system will have better results than beginners who use a 2D laparoscopic system. Compare the short-term outcomes of three dimensional (3D) versus two dimensional (2D) laparoscopic procedures used for cholecystectomy.

## METHODS

This study was conducted at minimally invasive surgery center of LUMHS Jamshoro, from 15th May 2017 to 16th December 2017. This is a comparative study. Ethical approval was taken from the institutional review board (LUMHS/REC/O87 Dated: 13.09.2019). Two groups each comprising of seventy three patients were made. Inclusion criteria were patients diagnosed with cholelithiasis without any co-morbid or any complications. Patients having risk factors for inability to get access to gall bladder via laparoscope and in whom the chances of conversion to open cholecystectomy were greater were not included as part of study. Exclusion criteria included patients with co-morbid or patients having any complication. All patients were diagnosed cases of cholelithiasis without any complications. Patients in both groups had near similar characteristics. Informed consent was taken before including patients in the study.

One group of patients underwent cholecystectomy under 3D laparoscopy while other group underwent 2D laparoscopy. Surgeons included in the study were all well-trained having more than five years’ experience and those having low expertise in three dimensional laparoscopy were not included. The short-term outcome noted were intraoperative and postoperative complications, conversion to open, operative time, mortality and hospital stay. Visual strain and headache for the surgeon in three D laparoscopic cholecystectomy.

The sample size was estimated to be 146 patients. Sample technique was non-probability consecutive sampling. Data was entered in SPSS version 25.0. Frequencies and percentages for gender, BMI, operative finding, intra and postoperative complications were done. Mean ±SD were reported for age and duration of illness. Outcome was compared by chi square test. Level of significance was set at ≤0.05.

## RESULTS

A total of 146 patients were included in the study. Both groups comprised of 73 patients. Group-A consists of 62 females and 11 males whereas Group-B comprised of 58 females and 15 males.

In Group-A, eight patients had ages between 20-35 years, thirty six patients had ages between 36-50 years while 19 patients had age greater than 50 years with means age 36±4.2 years. In Group-B, 21 patients had age 20-35 years, 28 patients had age 36-50 years and 24 patients had age greater than 50 years with means age 41±3.7 years.

About 8% of patients in Group-A had gall bladder rupture whereas in Group-B 2% had gallbladder rupture. 15% of patients in Group-A had bleeding from liver bed whereas 5.4% from Group-B had bleeding from liver bed. CBD injuries were very rare in both groups. One patient from Group-A had minor CBD injury. No patient from either group had duodenal injury.

Operative time range was 30 minutes to 90 minutes in both groups. The mean time in 2D laparoscopic cholecystectomy was 35.90±13.10 minutes and 3D laparoscopic cholecystectomy was 41.30±12.96 minutes (p 0.026). Eye symptoms in 2D imaging, revealed that eye strain was significantly noted in 2D imaging when compared to 3D. In 3D imaging, the difficulty in refocusing from one distance to another was significant observed when compared to 2D.

Post-operatively two patients from Group-A had port site bleeding with no patient having this complication in Group-B. Six patients had port site infection in Group-A whereas only one patient from Group-B had this complication. No patient from either group had urinary retention or colonic injury. The duration of hospital was one to three days. It was longer in complicated gallbladder about three days, whereas majority patients were discharged within 24 hours.

## DISCUSSION

Three D laparoscopy offers better visualization of structures however it is also associated with a few adverse effects. In low- and middle-income countries, the economic burden associated with the use of 3D laparoscopy makes its access difficult. The robotic surgery is providing the three dimensional view but it is costly and one cannot afford it low and middle income countries.^7^

Our study reports that 3D laparoscopic procedures are associated with low incidence of intra-operative and post-operative complications. Koppatz et al showed no difference in conducting 3D laparoscopic procedures and 2D laparoscopic procedures. Surgeons concluded that 3D laparoscopy made them more satisfied with better visualization.^1^ Komaei et al conducted a systemic review comparing the efficacy of 3D laparoscopic procedures with 2D laparoscopic procedures. This systemic review included five studies, three of which conclude that 3D laparoscopic techniques reduced the time required for cholecystectomy while the rest of two studies concluded that there wasn’t any significant difference among the two modalities.^8^

**Table-I:** Demographic variable.

*Variable*	*Procedure*		*Means Ratio*

	2D	3D	
Gender			
<Female	62(84.93%)	58(79.45%)	Ratio
Male	11(15.06%)	15(20.54%)	Group-A 5.6:1 Group-B 3.8:1
Age			
20-35 years	18(24.65%)	21(28.76%)	Means Group-A 36±4.2 Group-B 41±3.7
36-50 years	36(49.31%)	28(38.35%)	
> 50 years	19(26.02%)	24(32.87%)	
BMI			
Underweight = <18.5	13(17.80%)	7(9.58%)	Means Group-A 26±2.7 Group-B 24±8.2
Normal weight = 18.5–24.9	35(47.94%)	38(52.05%)	
Overweight = 25–29.9	17(23.28%)	23(31.50%)	
Obesity = 30 or greater	08(10.95%)	5(6.84%)	
Duration of Illness			
≤ 6 months	14(19.17%)	18(24.65%)	-
>6 months	59(80.82%)	55(75.34%)	

**Table-II:** Operative, postoperative finding and visual symptoms.

*Variable*	*Procedure*		*P value*

	2D	3D	
Over all per Operative Finding			
Adhesions in calot’s triangle	11(15.06%)	13(17.80%)	
Severe & tight adhesions around gallbladder and calot’s triangle	9(12.32%)	16(21.91%)	
Obscured anatomy in calot’s triangle	12(16.43%)	9(12.32%)	
Intrahepatic gallbladder	7(9.58%)	5(6.84%)	
Adhesions around gallbladder	7(9.58%)	10(13.79%)	0.045
Empyema	6(8.21%)	3(4.10%)	
Mucocele	4(5.47%)	4(5.47%)	
Contracted gall bladder	9(12.32%)	8(10.95%)	
Anatomical variation	8(10.95%)	5(6.84%)	
Intraoperative Complications			
Gallbladder rupture	6(8.21%)	2(2.73%)	
Bleeding from liver bed	11(15.06%)	4(5.47%)	0.001
CBD injuries	1(1.36%)	0	
Duodenal injury	0	0	
Postoperative Complications			
Nausea	5(6.84%)	1(1.36%)	
Postoperative fever	3(4.10%)	0	
Urinary retention	0	0	0.004
Colonic injury	0	0	
Port site bleeding	2(2.73%)	0
Port site infection	6(8.21%)	1(1.36%)
Visual Symptoms			
Eye strain	22(30.13%)	6(8.21%)	0.011
Difficulty in refocusing	4(5.47%)	37(50.68%)	
Headache	0	4(5.47%)	

Velayutham et al compared the operative performance of 3D laparoscopic procedures with 2D laparoscopic performance while liver resection. He concluded that there was no difference in contralateral wedge resections, combined resections, amount of blood loss and post-operative complications. However, the operative time was significantly reduced with 3D laparoscopy.^9^

Fergo et al conducted a systemic review analyzing the effectiveness of 3D laparoscopic techniques with 2D laparoscopic techniques in abdominal surgeries. He concluded that 3D laparoscopic techniques are superior in performing abdominal surgeries.^10^

Curro et al concluded that the total time for completion of laparoscopic cholecystectomy was equal with 3D and 2D laparoscopic procedures among experienced surgeons. However, the time taken was significantly less among naïve surgeons.^11^ These findings are similar to the findings of Almeida et al who concluded that 3D laparoscopic techniques are beneficial for naive surgeons to better understand the relations of various structures.^12^ Bilgen et al have reported that 3D laparoscopic cholecystectomies are superior than 2D laparoscopic cholecystectomies.^13^

In the study of Koppatz H et al^1^ reported intraoperative complications were gallbladder rupture 7 (6.7%) cases in 3D while 10 (9.7%) cases in 2D, intraoperative minor bleeding 4 (3.8%) cases in 3D while 4 (3.9%) cases in 2D and bleeding from liver bed 2 (1.9%) cases in 3D while one (1.0%) case in 2D. However, the in our study observed were gallbladder rupture 6(8.21%) cases in 2D while 2(2.73%) cases in 3D and bleeding from liver bed 11(15.06%) cases in 4(5.47%) cases 3D. International study reported Visual symptoms and eye strain were significant in 2D (p < 0.01) and difficulty in refocusing from one distance to another was significant in 3D laparoscopic imaging (p < 0.05)^6^. Compared with our study almost same result were eye strain more common in 2D while difficulty in refocusing mostly observed in 3D group.

Three dimensional laparoscopy has been associated with fewer intra-operative and post-operative complications and a shorter hospital stay. However further risks and benefits associated with its use need more clinical trials. Hoe et al have concluded that both modalities have similar outcome.^14^ However European consensus conference concluded that 3D laparoscopy procedure reduce time in operating room and box training. It also reduced the perioperative complications in laparoscopic suturing procedure.^15^

A recent study comparing the 3D high definition with 4 K (ultra-high definition) imaging showed that there is no decrease in operating time and errors during laparoscopic cholecystectomy with 3D imaging system.^16^ Our research found that difficulty in re-imaging from one distance to another showed the importance of eye strain and headache in 3D imaging. While eye strain was found to be statistically significant in 2D. Comparing our results with study of El Boghdady M reported similar findings.^6^

Dunstan M et al has also reported that 3D laparoscopy did not reduce operating time or errors but reduced dissection time in Calot’s triangle and complex cases and gall bladder perforation.^16^

## CONCLUSION

In our study 3D laparoscopic procedures were found to have low incidence of intra-operative and post-operative adverse events. However, being from a third world country, the economic burden associated with 3D laparoscopy restricts its use to certain surgical units. Further studies particularly conducted at larger scale are required to confirm if it has benefits in various surgical techniques over two-dimensional laparoscopy specially in complex cases and cases done in narrow and confined spaces like pelvis.

### Authors’ Contribution:

**ARS** Conceived, data collection and manuscript writing.

**AAS** Editing and final approval.

**MA** Data collection, writing, accuracy and integrity of work.
